# Protein interaction explorer (PIE): a comprehensive platform for navigating protein–protein interactions and ligand binding pockets

**DOI:** 10.1093/bioinformatics/btae414

**Published:** 2024-06-25

**Authors:** Fabien Mareuil, Alexandra Moine-Franel, Anuradha Kar, Michael Nilges, Constantin Bogdan Ciambur, Olivier Sperandio

**Affiliations:** Bioinformatics and Biostatistics Hub, Département Biologie Computationnelle, Institut Pasteur, USR 3756 CNRS, 75015 Paris, France; Structural Bioinformatics Unit, Department of Structural Biology and Chemistry, Institut Pasteur, Université de Paris, CNRS UMR3528, 75015 Paris, France; Collège Doctoral, Sorbonne Université, 75005 Paris, France; Structural Bioinformatics Unit, Department of Structural Biology and Chemistry, Institut Pasteur, Université de Paris, CNRS UMR3528, 75015 Paris, France; Structural Bioinformatics Unit, Department of Structural Biology and Chemistry, Institut Pasteur, Université de Paris, CNRS UMR3528, 75015 Paris, France; Structural Bioinformatics Unit, Department of Structural Biology and Chemistry, Institut Pasteur, Université de Paris, CNRS UMR3528, 75015 Paris, France; Structural Bioinformatics Unit, Department of Structural Biology and Chemistry, Institut Pasteur, Université de Paris, CNRS UMR3528, 75015 Paris, France

## Abstract

**Summary:**

Protein Interaction Explorer (PIE) is a new web-based tool integrated to our database iPPI-DB, specifically crafted to support structure-based drug discovery initiatives focused on protein–protein interactions (PPIs). Drawing upon extensive structural data encompassing thousands of heterodimer complexes, including those with successful ligands, PIE provides a comprehensive suite of tools dedicated to aid decision-making in PPI drug discovery. PIE enables researchers/bioinformaticians to identify and characterize crucial factors such as the presence of binding pockets or functional binding sites at the interface, predicting hot spots, and foreseeing similar protein-embedded pockets for potential repurposing efforts.

**Availability and implementation:**

PIE is user-friendly and readily accessible at https://ippidb.pasteur.fr/targetcentric/. It relies on the NGL visualizer.

## 1 Introduction

Protein–protein interactions (PPIs) are integral to various biological functions (Stumpf *et al.* 2008), constituting a diverse and crucial element of cellular processes. The multifaceted roles of PPIs have positioned them as a credible and alternative reservoir of potential drug targets (Sperandio *et al.* 2010). Going beyond their significance in biological pathways, the 3D) structure of PPIs plays a paramount role, influencing both their biological function and the potential binding of small molecules. This concept, known as “ligandability,” underscores the essential need for a ligandable binding site within the target—a foundational principle in drug discovery (Pérot *et al.* 2010) that gains particular importance in the context of PPIs.

Extensive documentation affirms the substantial variability in numbers and sizes of PPI binding sites. The determination of their ligandability necessitates a thorough, case-by-case examination (Fuller *et al.* 2009, Kuenemann *et al.* 2016). Despite challenges arising from the lack of readily available ligands for PPIs, there exists an opportunity to explore alternative approaches for evaluating the feasibility of targeting these interactions with small-molecule inhibitors.

Over the years, numerous web-based tools have emerged to address various queries concerning PPI. One category of tools focuses on analyzing PPI networks for visualization, such as PIMA (Mathew and Sowdhamini 2016), OpenPIP (Helmy *et al.* 2022), Cytoscape (Majeed and Mukhtar 2023), Proteinarium (Armanious *et al.* 2020), and Cellmap (Dallago *et al.* 2017). These tools utilize graph-based algorithms to construct and interpret large networks of interacting molecules, aiding in the identification of functional modules, gene-based clustering, pathway detection, and understanding disease mechanisms.

Another category includes tools like SiteMap (Halgren 2009), DoGSite3 (Graef *et al.* 2023a), PLIC database (Anand *et al.* 2014), P2Rank (Krivák and Hoksza 2018), Coach, and Cofactor (Roy *et al.* 2012). They use geometric analysis to detect protein surface cavities, along with features like pocket descriptors and druggability predictions. Pockdrug (Borrel *et al.* 2015) and DoGSite3 (Graef *et al.* 2023a), for instance, offer algorithms for pocket estimation and druggability prediction, while PLIC database focuses on binding site clusters and COACH (Yang *et al.* 2013) provides ligand-binding site prediction.

Recently developed tools like PiMine (Graef *et al.* 2023b) and ProteinsPlus (Schöning-Stierand *et al.* 2022) offer unique functionalities such as alignment and similarity assessment of protein–protein interfaces and interactive analysis of protein-ligand binding interfaces.

The Protein Interaction Explorer (PIE) described in this paper introduces a novel web-based approach for analyzing protein structures, with a focus on heterodimer complexes. PIE facilitates the identification of functional binding sites, prediction of hot spots, and visualization of protein-ligand interactions. It offers 3D visualization of druggability and interactibility predictions, along with graphical representation of the “pocketome” derived from the IPPIDB database, distinguishing itself from existing PPI analysis tools.

Protein Interaction Explorer (PIE) introduced as a novel feature on our website and database iPPI-DB, innovates the exploration of PPIs. Drawing upon extensive structural data encompassing thousands of heterodimer complexes, including those with successful ligands, PIE uses a holistic approach by integrating various visualization tools into a unified platform facilitated by the NGL JavaScript package (Rose and Hildebrand 2015). This integration empowers users to effortlessly delve into each set of structural data, providing a comprehensive understanding of PPIs and their modulators.

Going beyond conventional structural data analysis, PIE introduces unique features such as the integration of detected pockets through VolSite (Desaphy *et al.* 2012), functional binding site predictions from InDeep (Mallet *et al.* 2022), calculated hot spots using FoldX (Guerois *et al.* 2002), the ability to overlay liganded protein chains onto their corresponding heterodimer complex, and the introduction of a novel metric for pocket similarity (Moine-Franel *et al.* 2024). Noteworthy is PIE's distinctive application of pocket similarity metrics, enabling researchers to navigate the PPI pocketome. This functionality allows for the discovery of potential protein partners through structural similarities, thereby facilitating assisted repurposing efforts.

As we delve into the intricate world of PPIs, these advanced functionalities offered by PIE open new avenues for researchers and bioinformaticians. The integration of diverse tools within PIE not only enriches the dataset but also provides a holistic view of the molecular landscape of PPIs and how some have been successfully targeted with small molecules. This innovative platform not only accelerates computer-aided drug design but also enhances our comprehension of molecular interactions, adding valuable insights to the evolving field of PPI research (https://ippidb.pasteur.fr/tutorials).

## 2 Materials and methods

### Protein selection

The chosen protein subset encompasses both heterodimer protein–protein complexes (HD—heterodimer) and monomers paired with ligands associated with these heterodimers (PL—protein/ligand). The criteria for inclusion involve precise Uniprot annotation (Bateman *et al.* 2023), with heterodimer complexes comprising exactly two molecules displaying distinct Uniprot annotations. Each molecule must consist of more than three residues. Ligands associated with PL complexes must meet specific criteria, including a minimum number of heavy atoms and exclusive inclusion of drug-like atoms. These ligands are positioned at or near the interface associated with the heterodimer. The interaction patch determination relies on Euclidean distance calculations between all atoms of the protein target and its partner, with the distance threshold set at 6 Angstroms (Å).

### Structure quality filters

A set of 3D structure quality filters was applied to the subset. Only structures determined through nuclear magnetic resonance, X-ray crystallography, or cryogenic electron microscopy (cryo-EM) were considered. For X-ray crystal and cryo-EM structures, selected structures exhibited a resolution equal to or <3.5 or 3 Å, respectively. Additionally, the difference between R-free and R-factor (for X-ray structures) and Fourier shell correlation (for cryo-EM structures) was equal to or <0.07 and 0.143, respectively. The 3D structures excluded any atoms with alternative locations at the protein–protein or protein–ligand interfaces.

### Preparation steps

Before pocket detection, any incomplete amino acids in the structures were repaired using FoldX (Guerois *et al.* 2002) (version 5) software. Heteroatoms (specifically for HD complexes) and water molecules were removed. Subsequently, both HD and PL complexes underwent protonation using GROMACS (version 2020) software (Van Der Spoel *et al.* 2005, GROMACS 2020 Source code).

### Pockets detection, filtration, and characterization

VolSite was utilized for the detection and characterization of pockets. Pockets were identified in both the protein target and its corresponding partner for HD complexes, treating the partner as the ligand in a sequential manner. For PL complexes, pockets were detected in the monomer. Post-detection, only pockets meeting specific criteria, such as having at least four probes of their negative image located at 1 Å or less from a partner atom (protein or ligand), were retained. This filtering excluded non-orthosteric (HD) or non-liganded (PL) pockets. Pockets within monomers containing ligands were further classified as allosteric if the ligand was positioned >1 Å away from at least four probes of the negative image of the associated-heterodimer pocket. Orthosteric pockets were categorized as either competitive or non-competitive based on the proximity of the ligand to its protein partner.

We categorized PL pockets into three primary types: orthosteric competitive (PLOC), orthosteric non-competitive (PLONC), and allosteric (PLA) pockets. PLOC pockets involve a direct competition between the ligand and the epitope of the protein partner within the heterodimer. In contrast, PLONC pockets contain ligands within orthosteric pockets that do not directly compete with the protein's epitope but may influence its function or conformation. Lastly, PLA pockets, positioned near the orthosteric binding pockets of a heterodimer, do not directly overlap with the orthosteric site but may induce allosteric effects.

A total of 89 pocket descriptors were computed with the VolSite software. Then, a set of 10 supplementary descriptors amalgamating attributes from those initially derived by VolSite was computed, to furnish a more nuanced depiction of pocket characteristics. These 10 amalgamated descriptors were determined in accordance with the methodology outlined in Kuenemann *et al.* (2016). Finally, 10 more geometric descriptors were computed using the RDKit3D module, to encompass key pocket properties such as asphericity, sphericity index, molecular eccentricity, inertial shape factor, radius of gyration, principal moments of inertia, and normalized principal moments ratio. To keep consistency, these latter descriptors were specifically calculated using the MOL2 files of the VolSite pockets.

### Pocket similarity evaluation

The similarity between two pockets was assessed based on their Euclidean distance in the high-dimensional space of pocket properties (descriptors) calculated with VolSite. For each pocket, a set of 109 descriptors was initially computed and further reduced to 82 by keeping only those with non-zero values for >95% of the sample. Finally, all descriptors were re-scaled to zero mean and unit variance to reduce covariance effect in the distance calculation. Pairwise Euclidean distances between each pocket were computed, and a Gaussian kernel was applied to transform the distances into probabilities between 0 and 1, following Equation 1:

(1) $${\mathrm{PSI}}_{ij}=\exp\left(\frac{-d_{ij}^{2}}{2\sigma^{2}}\right)$$

where *d_ij_* is the Euclidean distance between pockets *i* and *j* (*i*⧣*j*) and σ is the standard deviation of d across all pairs.

### Pocketome visualization

The pocketome is visually represented as a minimum spanning tree using the TMAP (Probst and Reymond 2020) tool. This method selectively chooses pairs from the complete PSI matrix to span the entire dataset optimally, minimizing the total distance. The resulting “tree” provides a simplified yet powerful visual representation reflecting local proximity (pocket similarity) in the high-dimensional pocketome space.

### Hot spot prediction

FoldX (version 5) software was used to assess the impact of mutations at the interface on the stability of protein structure complexes. Residues exhibiting a change in free energy of 1.5 kcal/mol or more were identified as critical hot spots, while those with a free energy shift within the range of 0.5 to 1.5 kcal/mol were labeled as hot spots.

### Functional binding site prediction

InDeep was utilized for predicting functional binding sites. This tool anticipates ligand-binding sites, specifically targeting ligandable binding sites for iPPIs (inhibitors of PPIs) and epitope-binding interactability patches. The predictions are based on deep learning techniques and leverage a meticulously curated protein dataset to ensure accuracy and reliability.

## 3 Results

Following the filtration and pocket detection processes, the protein subset comprises 4770 HD complexes with 18 266 orthosteric pockets and an additional of 4188 PL complexes with 4972 liganded pockets. Within ligandable binding sites, 1830 pockets are engaged by an allosteric ligand, 817 pockets are bound by an orthosteric non-competitive ligand, and 2325 pockets are occupied by an orthosteric competitive ligand. Altogether, the dataset encompasses 52 distinct families of PPIs.

We designed PIE to explore a new pocket-centered dataset and establish links with the compound-centered dataset. The pocket-centered web interface comprises two main parts:

### 3.1 Query part

**Figure 1.**
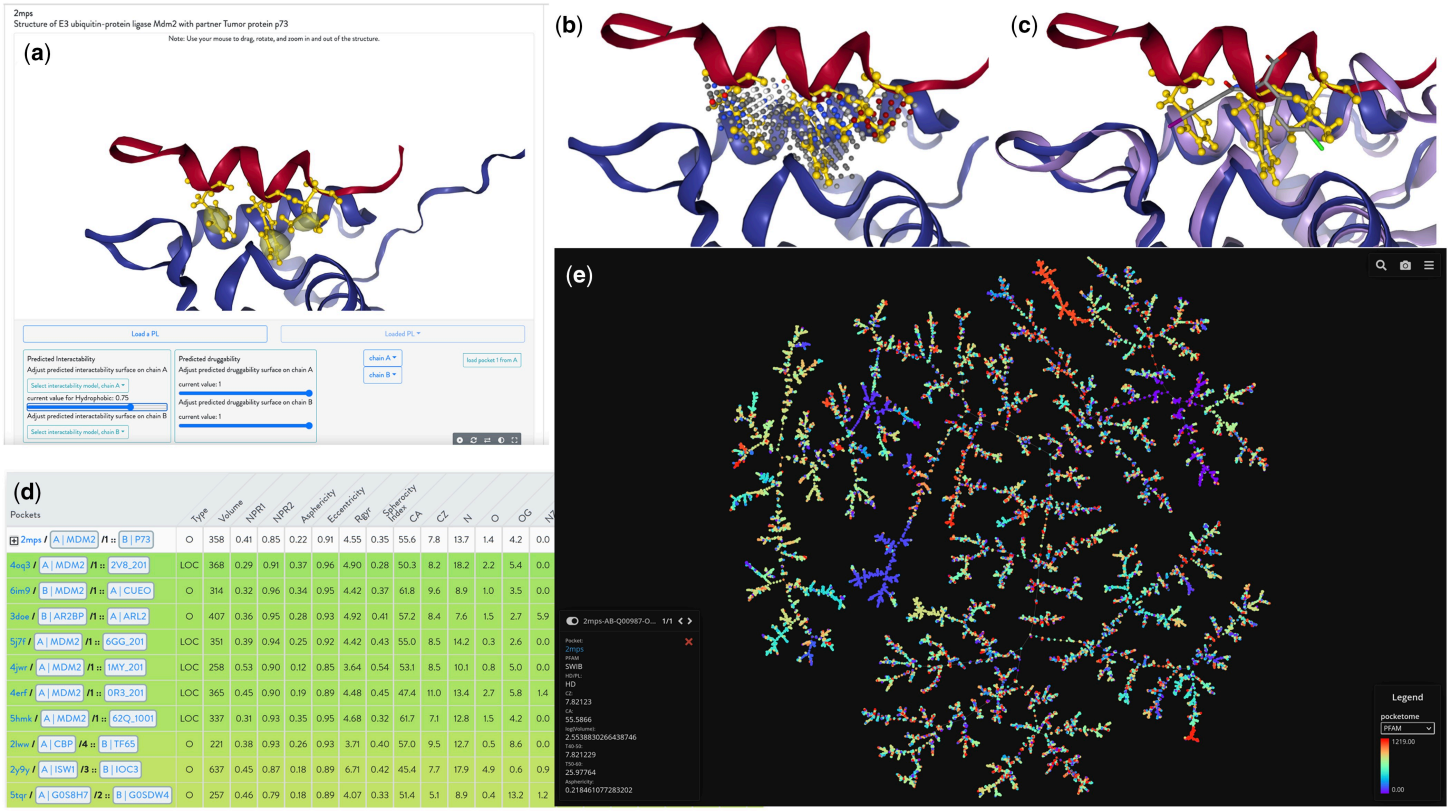
Visualization of the PDB code 2mps (HD) results from PIE in the iPPI-DB database. (**a**) Visualization of p53/MDM2 (pdb code 2mps) in the NGL javascript window. (**b**) VolSite pocket is presented as a negative image of the binding site. (**c**) Overlay onto the MDM2/p53 complex structures all available ligands for MDM2. (**d**) table beneath the GUI lists all identified neighboring pockets using PSI metrics. (**e**) showcases the full PPI pocketome visualization as a minimum spanning tree.

- Two search modes (basic and advanced) are available for users to conduct searches using criteria such as PDB ID, ligand ID, protein name, or organism.
- Results are displayed in a list format, sorted by the number of matches. The first result is loaded in the visualization part.
- The second section includes a constructed minimum spanning tree (TMAP) representing the pocketome (Fig. 1). This TMAP provides a simplified yet powerful visual representation, capturing pocket similarity in the high-dimensional pocketome space. Users can apply color-coding based on various options, such as Pfam identifier, dataset classification (HD or PL), pocket volume, exposure, hydrophobicity, aromaticity, and pocket asphericity. Each point on the TMAP represents a pocket, and the search bar allows users to focus on a specific pocket. Users can fly over each pocket one by one and click on a pocket to load the pocket-centered web interface of the related PDB.

### 3.2 Visualization part

- Developed with the NGL JavaScript package, the visualization part displays a 3D view of the protein complex (Fig. 1).
- Visualization options allow users to view pockets detected by VolSite, view ligands, change protein representation, and adjust the threshold for intractability and druggability surfaces prediction (InDeep).
- Within the available choices for depicting the 3D structures of HD and PL complexes, users can observe critical and warm hotspots residues detected by FoldX.
- At the bottom of the page, a table lists the main descriptors of each pocket detected by VolSite. Users can display the nearest pocket neighbors, and the distance between pockets is based on descriptors.
- The top five results from the HD and PL datasets are displayed. Links are available to allow exploration of the dataset, both compound-centered and pocket-centered, with proteins and ligands present in the table.
- Superimposition of all available PL complexes onto the chosen HD complexes. This feature can be used to investigate how the ligands were developed to modulate a given target, where is in the pocket they bind and with which possible hot spot they interfere.

### 3.3 Case study: exploring MDM2 structure interactions with PIE features

When utilizing the MDM2 structure bound by p53 (PDB code: 2mps) as the query, PIE's capabilities can be effectively assessed (see Fig. 1). First, the visualization of this system in the NGL javascript window (panel a) can be supplemented by FoldX hot spot predictions (yellow sticks) and our InDeep predictions. InDeep predictions, particularly those related to Interactability, are illustrated with yellow isolevel probabilities. Notably, the isolevel probabilities are adjustable using a slider that ranges from 0 to 1. This feature enables the precise highlighting of hydrophobic hot spots within the three-finger pharmacophore, offering a nuanced and customizable exploration of the hydrophobic channels depicted in the analysis. This nicely confirm the well-established three-finger pharmacophore on p53 (F306-W310-L313) bound to the MDM2 pocket.

Second, the VolSite pocket is presented as a negative image of the binding site (Panel b), featuring a set of probes pharmacophoric probes (aromatic, hydrophobic, etc.) which also nicely overlay with the three-finger pharmacophore. Then, the user can overlay onto the MDM2/p53 complex structures all available ligands for MDM2 (Panel c) whose structure has been solved, deposited in the PDB and kept in PIE. This allows a useful visualization of the binding modalities of the different ligands and how the chemical moieties of the ligands have been used to mimic the p53 epitope.

A table beneath the GUI lists all identified neighboring pockets using PSI metrics (Panel d). PSI ranges from 0 (depicted in red) to 1 (in green), with higher PSI values indicating greater pocket similarity. Notably, the nearest neighbors of 2mps' sole pocket include other MDM2 structures, as expected. Surprisingly, non-MDM2 systems, such as histone lysine acetyltransferase CREBBP bound to partner transcription factor p65 (PDB code: 2lww), WD_REPEATS_REGION domain-containing protein bound to SET domain-containing protein (PDB code: 5trq), and ADP-ribosylation factor-like protein 2 bound to ADP-ribosylation factor-like protein 2-binding protein (PDB code: 3doe), also emerge, sharing commonalities with the p53 helix that binds the MDM2 pocket with hydrophobic residues. Moreover, some of these systems, despite lacking alpha helices, exhibit a similar hydrophobic set of bound residues.

Panel e showcases the full PPI pocketome visualization, allowing for exploration and analysis of PPI pockets either by clicking on each pocket or using the search field. It becomes evident that the TMAP visualization of the pocketome provides an alternative approach for assessing results, considering the number of shared neighbors to establish proximity within the tree. The use of Pfam-based (Mistry *et al.* 2021) coloring further highlights the effectiveness of this categorization.

## 4 Discussion

Tools like PIE could play a pivotal role in bioinformatics and structural biology by enabling researchers to explore, analyze, and understand the intricate world of protein structures and interactions. Its significance lies in its capacity to bridge the gap between structural data and biological insights, ultimately contributing to advancements in drug discovery, disease understanding, and the broader field of molecular biology.

PIE is a valuable tool for addressing the challenges associated with studying PPIs and ligand interactions. Here are some ways in which PIE helps overcome these challenges:

- Data integration and accessibility: PIE promotes open data access and sharing. This is crucial for advancing PPI and ligand interaction research, as it allows researchers to validate findings, collaborate, and build upon existing knowledge. This comprehensive data integration allows researchers to access a wide range of information about PPIs and ligand interactions in one place, simplifying the process of data gathering and analysis.
- Visualization: PIE provides interactive and intuitive visualizations of protein structures and interactions. Visualization is crucial for understanding complex PPIs and ligand interactions, as it allows researchers to see the spatial arrangement of proteins and ligands, identify binding sites, and explore structural details (pockets, hot spots, and ligands).
- User-Friendly interface: A user-friendly interface and accessibility of PIE make it easier for researchers from diverse backgrounds to utilize the tool effectively. This inclusivity promotes collaboration and knowledge sharing in the scientific community, through persistent links.
- Comparative analysis: PIE enables comparative analysis of PPIs and ligand interactions across different proteins and complexes. Researchers can identify common binding motifs, structural similarities, and shared partners, which can lead to insights into conserved interaction patterns.
- Ligandability assessment: PIE incorporates annotations to identify binding sites, and druggable regions within proteins through InDeep predictions. This aids in prioritizing targets for further experimental validation and drug discovery efforts, as it helps researchers identify targetable sites on proteins and prioritize them for further investigation.
- Hot spot identification: PIE includes the prediction of hot spots, which are crucial for comprehending the fundamental factors that influence the affinity of protein interactions, and these predictions can provide valuable insights for drug design and therapeutic targeting.

In summary, PIE addresses the challenges associated with studying PPIs and ligand interactions by providing a multifaceted platform that combines structural and functional insights. This resource facilitates research in the fields of drug discovery, molecular biology, and structural biology by providing valuable insights into complex protein interactions.

## 5 Conclusion

PIE serves as a powerful resource that empowers researchers in bioinformatics and structural biology to explore, analyze, and understand the intricate world of protein structures and interactions. Its user-friendly interface, predictive tools, data integration, and customization options make it an essential asset for advancing research in these fields and accelerating discoveries that have a profound impact on fields such as drug development, disease understanding, and molecular biology.

PIE has the potential to significantly impact our understanding of molecular interactions and protein functions by providing researchers with a comprehensive toolkit to analyze and visualize complex interactions and ultimately have a deeper comprehension of the molecular basis of life.

## Data Availability

The dataset is available at https://zenodo.org/records/10227621 with the DOI https://doi.org/10.5281/zenodo.10227621 under a CC BY 4.0 licence.
